# Sexual selection

**DOI:** 10.1093/emph/eoz007

**Published:** 2019-02-13

**Authors:** Tim Janicke, Edward H Morrow

**Affiliations:** 1 Centre d’Écologie Fonctionnelle et Évolutive, UMR 5175, CNRS, Université Paul-Valéry Montpellier, École Pratique des Hautes Études, France; 2 Evolution, Behaviour and Environment Group, School of Life Sciences, University of Sussex, Brighton, UK

## DEFINITION AND BACKGROUND

Modern theory defines sexual selection as arising from competition for mating partners and/or for fertilizing the partner’s gametes. It incorporates Darwin’s original ideas of competition for and attraction of mating partners, plus recent insights that competition and choice can continue after mating. Sexual selection is generally strongest in males but there is a great diversity of sex roles across the tree of life, including reversals [1]. Sex differences in sexual selection often coincide with contrasting mating interests or optimal phenotypes of males and females i.e. sexual conflict. Both sexual selection and sexual conflict are potent forces driving trait evolution. Even though humans lack elaborate secondary sexual characters, sexual selection is still thought to operate in our species [2]. Both sexes exert significant choice when choosing a partner; notwithstanding cultural constraints, women tend to rate signals of status and resources more than men do, whereas men’s choice is primarily based on cues indicating youth and fecundity [2, 3].

## EXAMPLES IN CLINICAL MEDICINE AND PUBLIC HEALTH

Sexual selection can have a profound impact on the epidemiology of sexually transmitted diseases (STDs). For instance, polygynous populations are likely to show a higher incidence of STDs compared with monogamous ones. Moreover, stronger sexual selection in one sex means that only the most attractive individuals are successful in mate acquisition, leaving some individuals unmated. Such a skewed mating success may lead to lower prevalence of STDs in that sex.

Importantly, stronger sexual selection on males is expected to manifest in sex-specific resource allocation strategies, with males being selected to invest relatively more resources into mate acquisition at the expense of health and survival compared with females [4]. This could manifest as greater risk taking behaviour and accidents in men (especially in young adults).

Thus, sexual selection can give rise to sex differences in the susceptibility to diseases and in immune response [5]. On an evolutionary scale, strong sexual selection can purge deleterious alleles in a population more efficiently allowing more rapid evolution of resistance to new pathogens. Overall, stronger sexual selection in males in our early hominid ancestors together with constraints in the genetic architecture imposed by sexual conflict [6] might explain the sex-bias and persistence of pathogenic phenotypes in contemporary human populations. There are many examples of genetic variants having sex-specific effects on complex traits or disease predisposition. Clinical approaches need to account for the ubiquitous sex differences in disease profiles and risk factors as well as in the effectiveness of therapies for both sexes.
